# New therapeutic targets for endometriosis predicted through mendelian randomization analysis and case-control trials

**DOI:** 10.3389/fgene.2025.1631446

**Published:** 2025-08-15

**Authors:** Linyao Zheng, Yue Yin, Xiaotong Wang, Baoju Wang, Ran Cui, Guangmei Zhang

**Affiliations:** ^1^ Department of Gynaecology, The First Affiliated Hospital of Harbin Medical University, Harbin, China; ^2^ Department of Obstertrics, The Second Affiliated Hospital of Harbin Medical University, Harbin, China

**Keywords:** drug target prediction, endometriosis, plasma proteins, mendelian randomization, Rspo3

## Abstract

**Introduction:**

Endometriosis is a common chronic gynecological condition that affects approximately 10% of women of reproductive age worldwide.

**Methods:**

This study utilized large-scale genome-wide association study data and explored the causal relationship between blood metabolites, plasma proteins, and endometriosis via Mendelian randomization and colocalization analysis methods. Clinical pathological data were collected, and hypotheses were validated through experiments such as ELISA, RT-qPCR, and Western blotting.

**Results:**

*RSPO3* and *FLT1* were found to be potentially associated with endometriosis within the proteome. External validation and colocalization analysis confirmed the robustness of the association with *RSPO3*. Blood and tissue samples were collected from clinical patients to assess the accuracy of these predictions.

**Discussion:**

These results suggest that *RSPO3* may be a new target for the treatment of endometriosis, providing a direction for future drug development.

## 1 Introduction

Endometriosis (EM) is a common gynecological disease that affects approximately 10% of women of reproductive age worldwide (Zondervan et al., 2020). It causes chronic pelvic pain, menstrual pain and other symptoms that afflict women for a long time. Various treatment options, including medications, surgical interventions, and conservative treatment strategies, aim to alleviate symptoms and restore fertility (Csirzó et al., 2024). However, the existing treatment options are far from satisfactory. Surgical treatment cannot prevent the recurrence or slow the progression of the disease. Additionally, side effects such as contraception caused by drugs are not desirable (Saunders and Horne, 2021). Therefore, the development of therapeutic targets for endometriosis is needed. Genome-wide association studies (GWASs) with intermediate phenotypes, such as metabolite and protein level changes, provide functional evidence to map disease associations and translate them into clinical applications (Suhre et al., 2017). On the one hand, metabonomics is rich in information, which can reflect complex biochemical networks and respond more directly and sensitively to the disease state of an organism, making it an important candidate for understanding disease phenotypes (Liu and Locasale, 2017; Schrimpe-Rutledge et al., 2016; Zhang et al., 2012). The biological characteristics of metabolites from human biological fluids reveal the relationships among genotype, environment and phenotype, and are attractive biomarkers for clinical diagnosis, disease prognosis and classification (Qiu et al., 2023). Compared with upstream proteomics and genomics, metabolomics is characterized by its end-effect and amplification effect. On the other hand, protein abundance is highly variable, with expression patterns that are dependent on cell type and time, as well as posttranslational modifications. These factors carry biological information that cannot be obtained through genomics or transcriptomics (Wilhelm et al., 2014). Most of the targets of existing approved drugs are human proteins, and disease development is clearly correlated with protein expression and activity. Plasma proteins play key roles in a range of biological processes and are key targets for patent drug analysis (Santos et al., 2017). GWASs of plasma protein levels have identified genetic variants linked to proteins, providing an opportunity to explore potential drug targets through Mendelian randomization (MR) analysis. MR analysis employs genetic variants as instrumental variables (IVs) to reveal the relationships between risk factors (e.g., metabolites, circulating proteins) and outcomes. Compared with randomized controlled trials (RCTs), MR analysis can reduce bias by controlling for confounding factors, thus providing supporting evidence for the causal relationship between exposure and outcomes (Sanderson et al., 2022). We investigated the causal relationships between blood metabolites and plasma proteins and EM via a systematic two-sample MR analysis. In this study, we identified a potential association between the plasma protein RSPO3 and EM, and validated the prediction through experimental validation. These findings provide mechanistic insights into the development and expression of this disease, as well as opportunities for the development of new therapeutic targets.

## 2 Materials and methods

### 2.1 Study design

**FIGURE 1:**
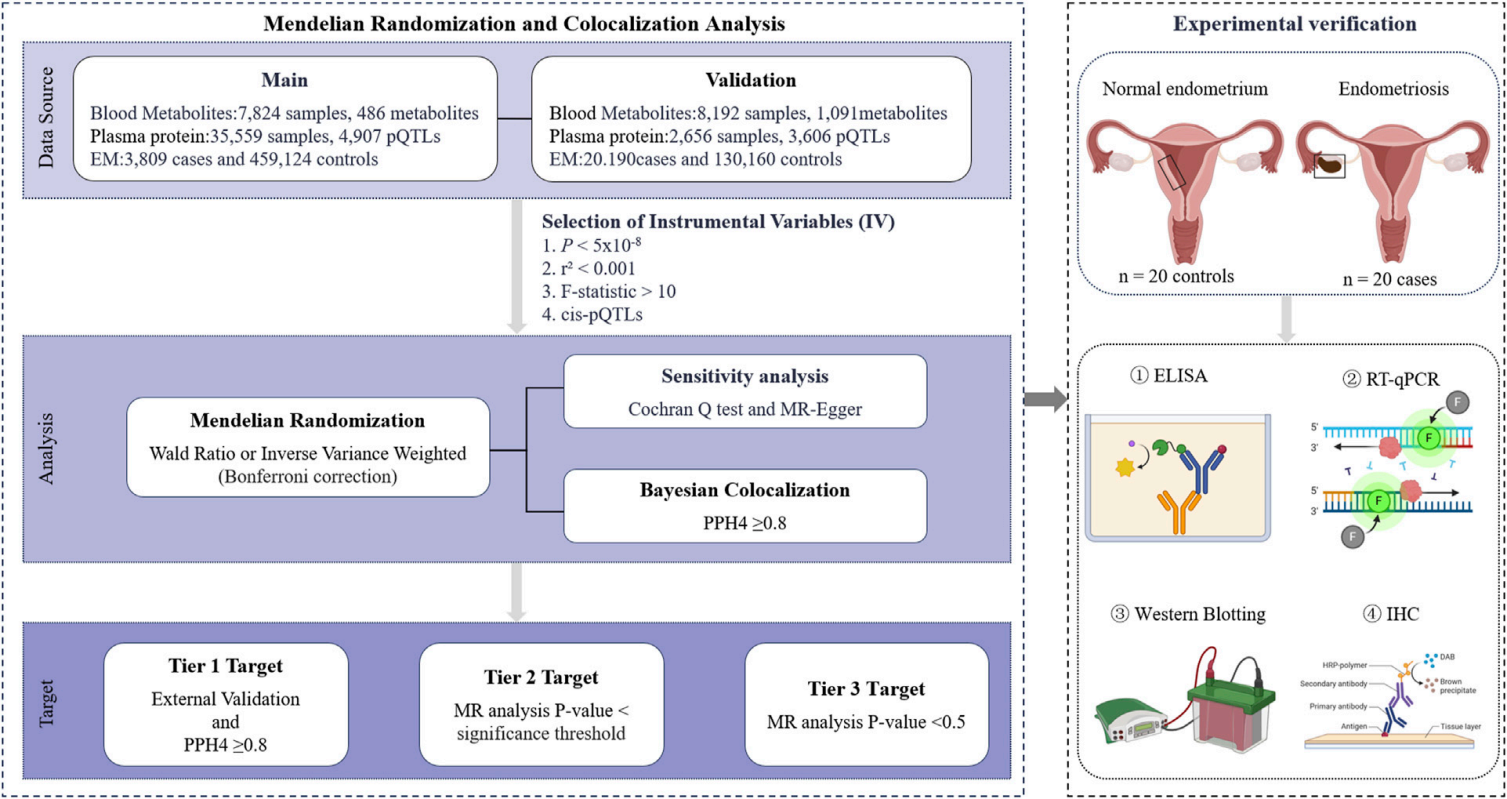
Study design. EM, endometriosis; cis-pQTLs, cis-protein quantitative trait loci; PPH4, posterior probability of hypothesis 4; ELISA, enzyme-linked immunosorbent assay; RT-qPCR, reverse transcription quantitative polymerase chain reaction; IHC, immunohistochemistry. Images were created by BioRender (https://app.biorender.com/).

The study design is shown in Figure 1. Publicly available large-scale GWAS related data sources have all received approval from the appropriate ethical review boards. Supplementary Table S1 lists the relevant GWAS data. The experimental verification part was managed and approved by the Ethics Committee of Harbin Medical University (KY 2022-155 at 28-06-2022).

### 2.2 Data sources of blood metabolites

For the primary analysis, we utilized summary-level data on blood metabolites from Shin et al. (Shin et al., 2014), encompassing 7824 samples from the German KORA F4 study and British Twins United Kingdom cohort (Krumsiek et al., 2012), covering a total of 486 metabolites. These data were accessed through the IEU OpenGWAS project (https://gwas.mrcieu.ac.uk/).

For the validation analysis, blood metabolite data were obtained from a publicly available dataset published by Chen et al. (Chen et al., 2023), which is accessible via the GWAS Catalog (https://www.ebi.ac.uk/gwas/), under accession numbers GCST90199621 to GCS90201020. To minimize population stratification bias, only individuals of European ancestry were included, ensuring comparable geographic and ancestral backgrounds (Lin et al., 2024). The final validation dataset comprised 1091 plasma metabolites and 309 metabolite ratios measured in 8192 European individuals.

### 2.3 Data sources of plasma proteins

For the primary analysis, we selected a large-scale GWAS of 35,559 Icelanders (Ferkingstad et al., 2021). This study utilized an aptamer-based multiplexed immunoaffinity assay (SOMAscan V4) to identify 4,907 cis-plasma protein quantitative trait loci (cis-pQTLs) associated with genetic variants. Detailed experimental procedures and the related GWAS data are available in the original publication (Ferkingstad et al., 2021). For the external validation analysis, plasma cis-pQTL data were obtained from a cohort study by Zheng et al. (2020). The researchers identified 3,606 pQTLs derived from 2,656 proteins across five GWASs (Suhre et al., 2017; Sun et al., 2018; Folkersen et al., 2017; Yao et al., 2017; Emilsson et al., 2018).

### 2.4 Data sources for endometriosis research

Summary-level data for the study outcomes are accessible from the United Kingdom Biobank (https://www.ukbiobank.ac.uk/) (Elsworth et al., 2020) and the FinnGen population database (https://www.finngen.fi/en) (Kurki et al., 2023). For the primary MR analysis, we obtained endometriosis GWAS data from the United Kingdom Biobank (ukb-b-10903, “self-reported: endometriosis”). This dataset comprised 3,809 cases and 459,124 controls.

To validate the metabolites and proteins identified in the primary analysis, we obtained aggregate data from the FinnGen R12 release. This integrated resource, comprising genome-wide and disease registry data, includes 20,190 cases and 130,160 controls. The median age was 37.27 years in cases and 38 years in controls. Crucially, there was no participant overlap between the United Kingdom Biobank and FinnGen data sources.

### 2.5 Selection of instrumental variables (IVs)

This study investigated the causal relationships between blood metabolites, plasma proteins as exposures, and EM as outcomes by adhering to the basic principles and core assumptions of MR analysis. To minimize bias and obtain more accurate results, two-sample MR was conducted under the following conditions: (1) Association assumption: the IVs are strongly correlated with exposure factors (i.e., blood metabolites and plasma proteins); (2) Independence assumption: the IVs are independent of outcome variables and confounding factors; (3) Exclusion assumption: the IVs can affect the outcome only through the exposure. Specifically, the selection of IVs meets the following criteria: (1) Genome-wide significant association: tool variables were selected on the basic of a significance threshold of *P* < 5 × 10^−8^ (Hemani et al., 2018); (2) No linkage disequilibrium (LD) clumping: r2 < 0.001; clump distance = 1 Mb. (3) To eliminate weak instrument bias, the F-statistic was used to estimate the strength of the instrumental variables. In the subsequent analysis, single nucleotide polymorphisms (SNPs) with F-statistics less than 10 were removed to obtain more robust instrumental variables (Holsinger and Weir, 2009). (4) A threshold of *P* = 0.05 was applied, and SNPs associated with the outcome were excluded from the subsequent analysis. In addition, in the selection of IVs, we chose genetic tools closely related to the target gene (cis-pQTLs) rather than those located further from the gene (trans-pQTLs) to minimize the potential violation of the exclusion restriction assumption (Ren et al., 2023).

### 2.6 Collection of clinical samples

The blood and lesion tissues (n = 20) of patients with endometriosis who underwent surgical treatment at the First Affiliated Hospital of Harbin Medical University were collected, and the an average age was 37 ± 6.4 years. In addition, blood and endometrial tissues from patients who underwent hysterectomy due to cervical lesions were collected as controls (n = 20). The control group had no endometrial-related diseases, with an average age of 46 ± 2.8 years. All patients included in the study were of childbearing age, had regular menstrual cycles, and fasted when blood samples were taken. The exclusion criteria were as follows: ① use of hormonal drugs within the last 6 months; ② placement of an intrauterine device (including the levonorgestrel intrauterine system); ③ history of malignant tumors. All the tissues were independently verified by two experienced pathology experts.

### 2.7 Enzyme-linked immunosorbent assay (ELISA)

The target protein concentration in the plasma was detected via a double-antibody sandwich ELISA method. The Human R-Spondin3 Elisa Kit purchased from BOSTER Biological Technology Co. Ltd., was used for the quantitative measurement of RSPO3 levels in the plasma of patients from both the EM and control groups. The samples were not diluted according to the manufacturer’s recommendations. The O.D. value was measured at 450 nm via a microplate reader, and the sample concentration was calculated.

### 2.8 Reverse transcription quantitative polymerase chain reaction (RT-qPCR)

To detect gene expression in tissues, total RNA was extracted using TRIzol reagent. After tissue lysis, chloroform (TRIzol: chloroform = 5:1) was added in proportion, followed by vortexing and centrifugation. The upper aqueous phase was transferred into another EP tube, and isopropanol was added before centrifuging for 10 min. The white pellet was washed with 75% ethanol, and after centrifugation, the pellet was air-dried at room temperature. The pellet was dissolved in an appropriate amount of DEPC-treated water. After the concentration was measured, cDNA was synthesized using *EasyScript* ^®^ One-Step gDNA Removal and cDNA Synthesis SuperMix (TransGen Biotech, China). mRNA expression was quantified via 2 × *PerfectStart* ^®^ Green qPCR SuperMix (TransGen Biotech, China), with GAPDH as the internal reference. The experiment was performed in triplicate, and relative expression levels were calculated via the 2^−ΔΔCT^ method. The primer sequences are listed in Table 1 and were purchased from Sangon Biotech (Shanghai, China).

**TABLE 1:** Sequences of Primers sequences used in this study.

Genes	Primer sequences
RSPO3	F: 5′-GAA​ACA​CGG​GTC​CGA​GAA​ATA-3′
	R: 5′-CCC​TTC​TGA​CAC​TTC​TTC​CTT​T-3′
GAPDH	F: 5′- GGA​GCG​AGA​TCC​CTC​CAA​AAT-3′
	R: 5′-GGC​TGT​TGT​CAT​ACT​TCT​CAT​GG-3′

### 2.9 Western blot (WB)

After the tissue was rinsed with cold physiological saline, it was minced and weighed. For every 20 mg of tissue, 100 μL of RIPA lysis buffer containing PMSF was added, and the tissue was homogenized in an ice bath for 1–2 min using a tissue grinder, followed by lysis on ice for 30 min. The mixture was then centrifuged at 12,000 rpm for 10 min at 4 °C, and the supernatant was collected. The protein concentration was determined via a BCA protein assay kit. Equal amounts of protein (20 μg) were separated by SDS-PAGE and then transferred to a PVDF membrane. The membrane was incubated overnight at 4 °C with RSPO3 (1:500, Invitrogen, USA) and β-tubulin (1:20,000, Proteintech, China). The membrane was then washed 6 times with TBST, for 5 min each time, and incubated with peroxidase-conjugated goat anti-rabbit IgG (H + L) (1:30,000, ZSGBbio, China) at room temperature for 1 h according to the manufacturer’s instructions. After washing, the membrane was developed with enhanced chemiluminescence (ECL) detection reagent (Biosharp, China), and the results were observed. The band density was quantified via ImageJ software for further statistical analysis.

### 2.10 Hematoxylin and eosin (HE) and immunohistochemistry (IHC) staining

The tissues were fixed with 4% paraformaldehyde at room temperature, dehydrated, and paraffin-embedded. After the wax blocks were frozen and solidified, the sections were cut, baked at 60 °C for 1 h, and subjected to gradient deparaffinization and rehydration via xylene and ethanol (100%–70%), followed by water. The sections were stained with hematoxylin, washed with water, and differentiated with a differentiating solution. After the addition of eosin staining solution, the sections were washed with water, dehydrated, cleared, and mounted. The slides were observed under a microscope, and images were taken and recorded. The original wax blocks were sectioned again, baked, deparaffinized, and rehydrated. Microwave radiation was used for antigen retrieval, and endogenous peroxidase activity was blocked with hydrogen peroxide. The sections were then blocked with 2.5% goat serum. After overnight incubation at 4 °C with the primary antibody (RSPO3, 1:200, Proteintech, China), the slides were washed three times with PBST. The secondary antibody was applied, and the samples were incubated at room temperature for 30 min, followed by three washes with PBST. DAB chromogenic reactions and hematoxylin staining were performed, and the slides were mounted with resin. Images were collected and processed.

### 2.11 Statistical analysis

#### 2.11.1 Mendelian randomization analysis

All the statistical analyses were performed in the R program. Two-sample MR analysis was performed by index SNPs of the metabolome and proteome to capture the associations between them and EM. This process is achieved via “TwoSampleMR” package (https://github.com/MRCIEU/TwoSampleMR). Effect estimates were generated via Wald ratios when exposures detected by individual SNPs were considered. When there are multiple SNPs, the weighted average of the rate estimates is used, and the weighted rate is the inverse variance weighted (IVW) of the rate estimates (Deng et al., 2022). To minimize the possibility of false positives, Bonferroni correction was used to solve the problem of multiple comparisons. We then adopted the significant variation strategy, that is, after replacing the GWAS data source, we reperformed rigorous MR analysis using genome-wide significant SNPs as a genetic tool to verify the preliminary findings (Lin et al., 2023).

#### 2.11.2 Sensitivity analysis

To verify the stability of the research results, the Cochrane Q method was used for heterogeneity analysis. When *P* > 0.05, there was no heterogeneity (Burgess et al., 2013). Additionally, the MR-Egger method was used to detect the presence of bias in potential genetic tools (Burgess and Thompson, 2017), and the *I* ^*2*^_*_GX*_ statistic was calculated. An *I* ^*2*^_*_GX*_ > 90% was considered to indicate high accuracy of the MR-Egger method (Bowden et al., 2016).

#### 2.11.3 Bayesian colocalization analysis

Colocalization analysis is used to identify whether two traits are driven by causal variants in the same region. We performed genetic colocalization analysis using the “coloc” package (https://github.com/chr1swallace/coloc) (Giambartolomei et al., 2014) with linkage disequilibrium (LD) correction. This method tests the posterior probability of shared variants between plasma proteins and endometriosis, and includes five hypotheses: H0: no association with either trait; H1: associated only with trait 1; H2: associated only with trait 2; H3: associated with both traits but driven by different variants; H4: associated with both traits and driven by the same variant. When the posterior probability of Hypothesis 4 (PPH4) is ≥0.8, there is considered significant colocalization between the two signals (Foley et al., 2021).

#### 2.11.4 Clinical sample statistical analysis

Statistical analysis was performed via the GraphPad 10.1.2 program, with all data presented as the means ± standard errors of the means (SEMs). Data following a normal distribution were compared via Student’s t-test, whereas nonparametric tests were used for data that did not follow a normal distribution. *P* < 0.05 was considered statistically significant.

## 3 Results

### 3.1 Metabolome-wide MR analysis

**FIGURE 2:**
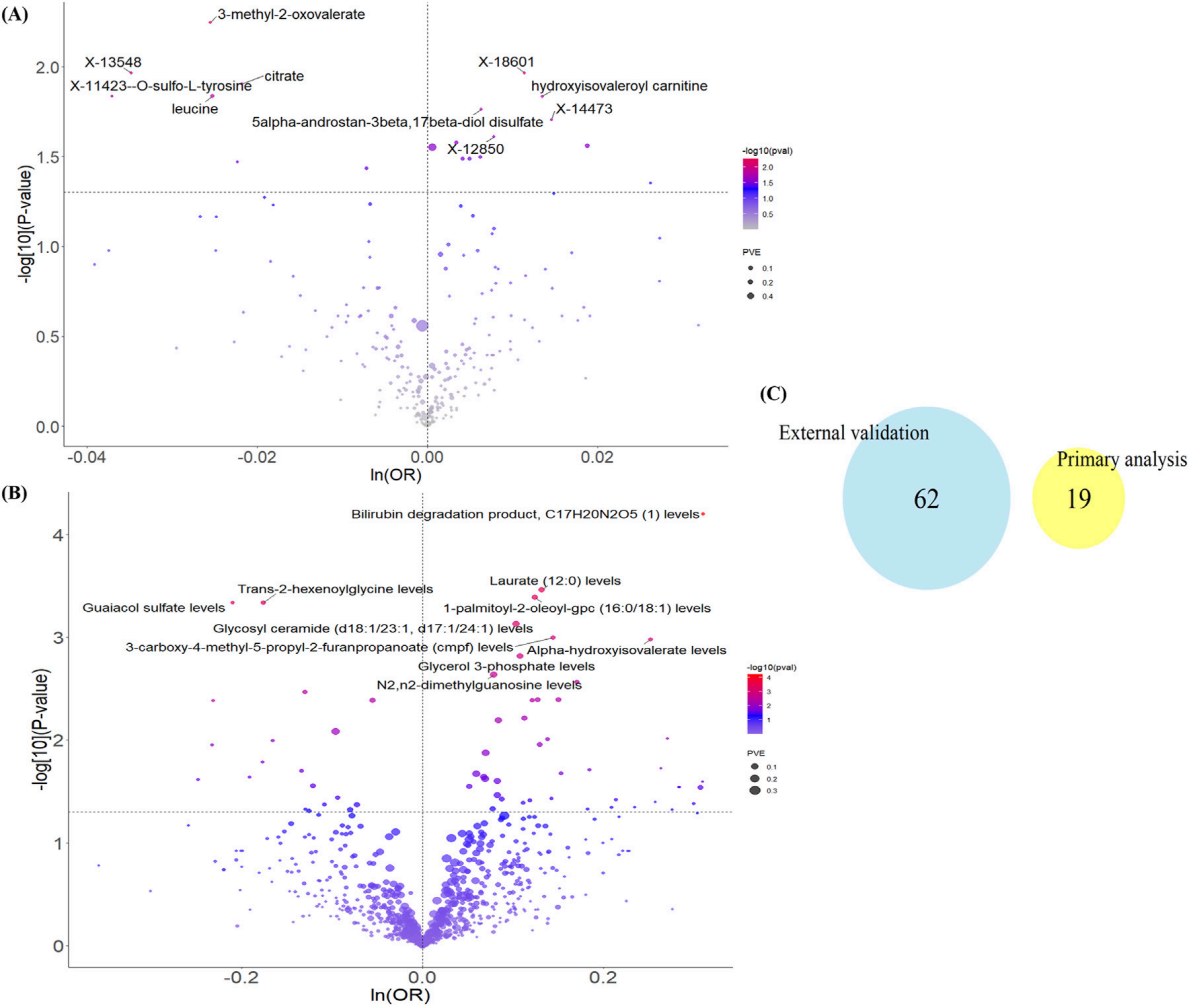
Results of Mendelian randomization analysis of blood metabolites and endometriosis. Volcano plots of causality between **(A)** 486 blood metabolites in the main analysis and **(B)** 1400 blood metabolites in the external validation and EM, with only the 10 most significantly different metabolites labeled. **(C)** Venn diagram of the two sets of results. ln: natural logarithm; PVE: proportion of variance explained.

As described, we adhered to the core principles and assumptions of MR analysis and applied strict criteria for instrumental variable selection. In the primary MR analysis, we utilized 486 metabolites from the Shin et al. study as exposures and endometriosis status (3,809 cases vs. 459,124 controls) from the United Kingdom Biobank as the outcome. The results identified 19 metabolites with a potential causal relationship with EM (P < 0.05). These included metabolites associated with increased EM risk (e.g., hydroxyisovaleroyl carnitine, 5alpha-androstan-3beta, 17beta-diol disulfate) and metabolites associated with decreased EM risk (e.g., 3-methyl-2-oxovalerate, citrate, leucine). However, none of these associations remained statistically significant after Bonferroni correction (Figure 2A; Supplementary Table S2).

For external validation, we replaced the data sources for both exposures and outcomes. Specifically, exposures comprised 1091 plasma metabolites and 309 metabolite ratios from the Chen et al. study, and the outcome was endometriosis status (20,190 cases vs. 130,160 controls) from the FinnGen R12 release. Applying the same MR methodology, the results indicated 62 metabolites with a potential causal relationship with EM. Again, none survived Bonferroni correction (Figure 2B; Supplementary Table S2). Furthermore, comparing the 19 metabolites identified in the primary analysis with the 62 from the validation analysis revealed no overlap (Figure 2C). This suggests that currently known blood metabolites may not robustly promote EM occurrence, or that their effects are context-dependent.

### 3.2 Proteome-wide MR analysis

**FIGURE 3:**
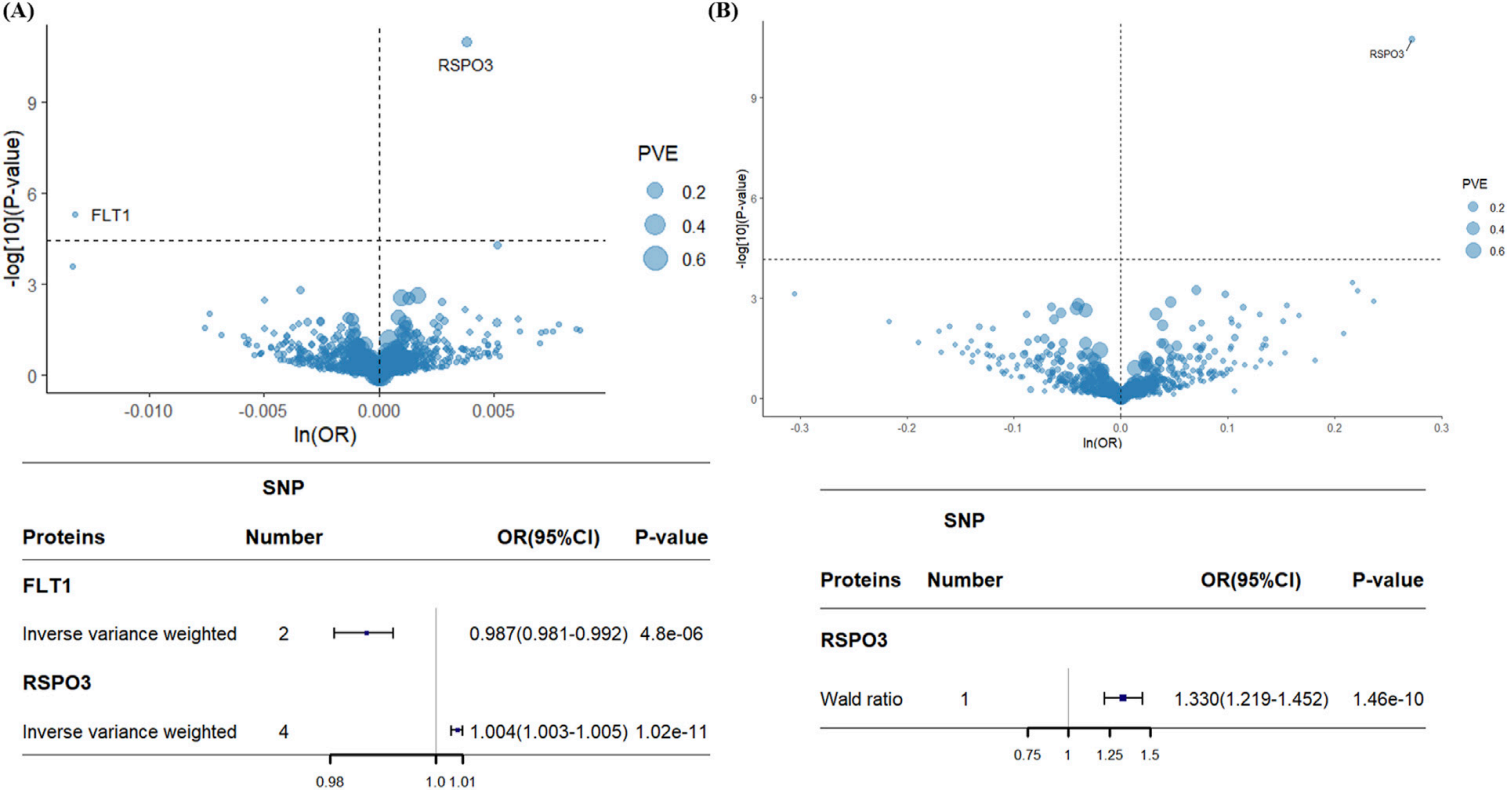
Volcano plots and forest plots for associations of genetically predicted plasma protein levels with EM in MR analysis. **(A)** Association between 1394 plasma proteins and EM. **(B)** Association between 732 plasma proteins and EM. Tagged genes refer to MR findings after Bonferroni correction. The OR for an increased risk of EM refers to an increased plasma protein SD. ln: natural logarithm; PVE: proportion of variance explained; OR: odds ratio; 95% CI: 95% confidence interval.

Following strict IVs selection, 4,144 SNPs corresponding to 1,394 proteins met the inclusion criteria. Two-sample MR analysis identified 57 proteins with a potential causal association with EM (P < 0.05; Supplementary Table S3). To account for multiple comparisons, we applied Bonferroni correction, setting a significance threshold of 0.05/1394 (*P* < 3.59 × 10^−5^). After correction, only FLT1 and RSPO3 remained significantly associated with EM (Figure 3A). Genetically predicted decreases in FLT1 levels and increases in RSPO3 levels were associated with higher EM risk. Specifically, for each 1-SD increase in genetically predicted protein levels, FLT1 had an odds ratio of 0.987 (95% CI = 0.981–0.992; *P* = 4.8 × 10^−6^), and RSPO3 had an odds ratio of 1.004 (95% CI = 1.003–1.005; *P* = 1.02 × 10^−11^).

For external validation, we screened 732 proteins with 736 IVs. After performing two-sample MR analysis (Supplementary Table S4) and applying Bonferroni correction, increased genetically predicted RSPO3 levels were again significantly associated with elevated EM risk (OR = 1.31; 95% CI = 1.21–1.42; *P* = 1.7 × 10^−11^), consistent with the primary analysis (Figure 3B). However, the association between decreased FLT1 levels and increased EM risk observed in the primary analysis was not replicated in the validation cohort.

To rule out the possibility that the non-significance of FLT1 in external validation was related to population stratification, we conducted a comparative analysis of allele frequencies for FLT1-associated SNPs between United Kingdom Biobank and FinnGen cohorts. As presented in Table 2, the allele frequencies of FLT1-associated SNPs demonstrated minimal differences between the two cohorts (maximum ΔAF = 0.012), which was substantially below the critical threshold of 0.1. These findings confirm genetic homogeneity between the cohorts and suggest that population stratification is unlikely to have influenced our results.

**TABLE 2:** The allele frequencies of FLT1-Associated SNPs between United Kingdom biobank and FinnGen.

SNP^a^	UKB_AF^a^	FinnGen_AF	ΔAF
rs56728557	0.1413	0.1536	0.0123
rs34867831	0.1347	0.1250	0.0097

^a^ SNP, single nucleotide polymorphism; AF, allele frequencies.

A sensitivity analysis was performed on the results of the main analysis to test the reliability of the MR analysis results. Through the Cochran Q test, we identified no evidence of heterogeneity in the proteins we assessed (*P*_*Q-test*_ > 0.05) (Table 3). Additionally, we calculated the *I* ^*2*^ statistic to mitigate the power limitation caused by the small number of SNPs. For this analysis, we calculated the *I* ^*2*^ statistic using the formula *I* ^*2*^ = max (0% [(Q - df)/Q] × 100%). The calculation showed that the *I* ^*2*^ values for both FLT1 and RSPO3 were 0%, further confirming minimal heterogeneity among the instrumental variable estimates. Since only one variant source was involved in the external validation of RSPO3, a heterogeneity test could not be conducted.

**TABLE 3:** Analysis of heterogeneity in plasma protein main analysis results.

Exposure	Outcome	Method	Cochran’s Q	Q_df	Q_pval
FLT1	UKB-B-10903	IVW^a^	0.2240	1	0.6360
RSPO3	UKB-B-10903	IVW	0.1908	3	0.9791

^a^ IVW, inverse variance weighted.

**FIGURE 4:**
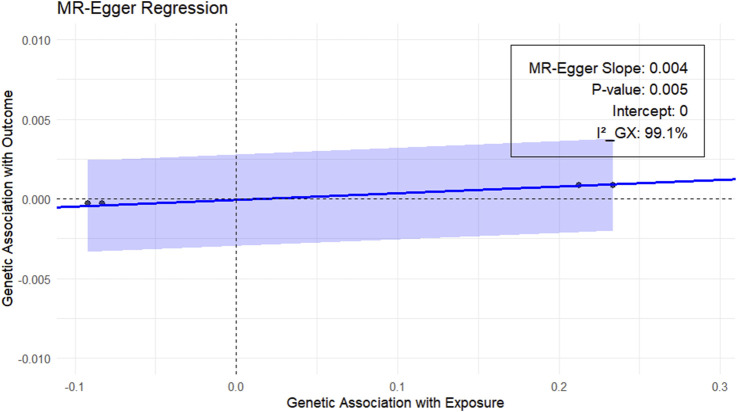
MR-Egger analysis of the plasma proteins RSPO3 and EM (UKB-B-10903). Blue regression line: MR-Egger regression line; gray shaded part: 95% confidence interval of the regression line.

Next, we performed the MR-Egger test to clarify whether the results were biased. Because more than two genetic variants were required for the MR-Egger test, only RSPO3 in the main analysis was used as the exposure. The results are shown in Figure 4, with an MR-Egger intercept of 0 and an *I* ^*2*^_*_GX*_ of 99.1%, indicating reliable results.

### 3.3 Colocalization analysis of pathogenic proteins

Bayesian colocalization analysis of plasma proteins and EM was performed to investigate the genetic signals shared between them. We tested the causal effects of the identified plasma proteins in EM. The results showed that RSPO3 exhibited significant colocalization with EM (PPH4 ≥ 0.8), whereas FLT1 shared the same causal variant with EM with a low probability (Table 4). Notably, while both RSPO3 and FLT1 showed significant MR associations, their colocalization results differed substantially - RSPO3 exhibited high PPH4 (0.802/1.000) indicating shared causal variants, whereas FLT1 showed low PPH4 (0.021) suggesting distinct underlying genetic architectures. This distinction reflects fundamental differences in their respective loci: RSPO3’s association appears driven by a single causal variant with strong LD, while FLT1’s association likely involves multiple independent variants with weaker LD patterns. Experimental verification of differential expression of RSPO3 in EM.

**TABLE 4:** Colocalization of plasma proteins and endometriosis.

Outcome	Exposure	SNP^a^	F_statistics	PPH4^a^
UKB-B-10903	RSPO3	rs853974	102.706	0.802
		rs9285458	849.828	
	FLT1	rs34867831	29.788	0.021
		rs56728557	32.809	
FinnGen	RSPO3	rs2489623	121.449	1.000

^a^ SNP, single nucleotide polymorphism; PPH4, posterior probability of Hypothesis 4.

### 3.4 Experimental verification of differential expression of RSPO3 in EM

**FIGURE 5:**
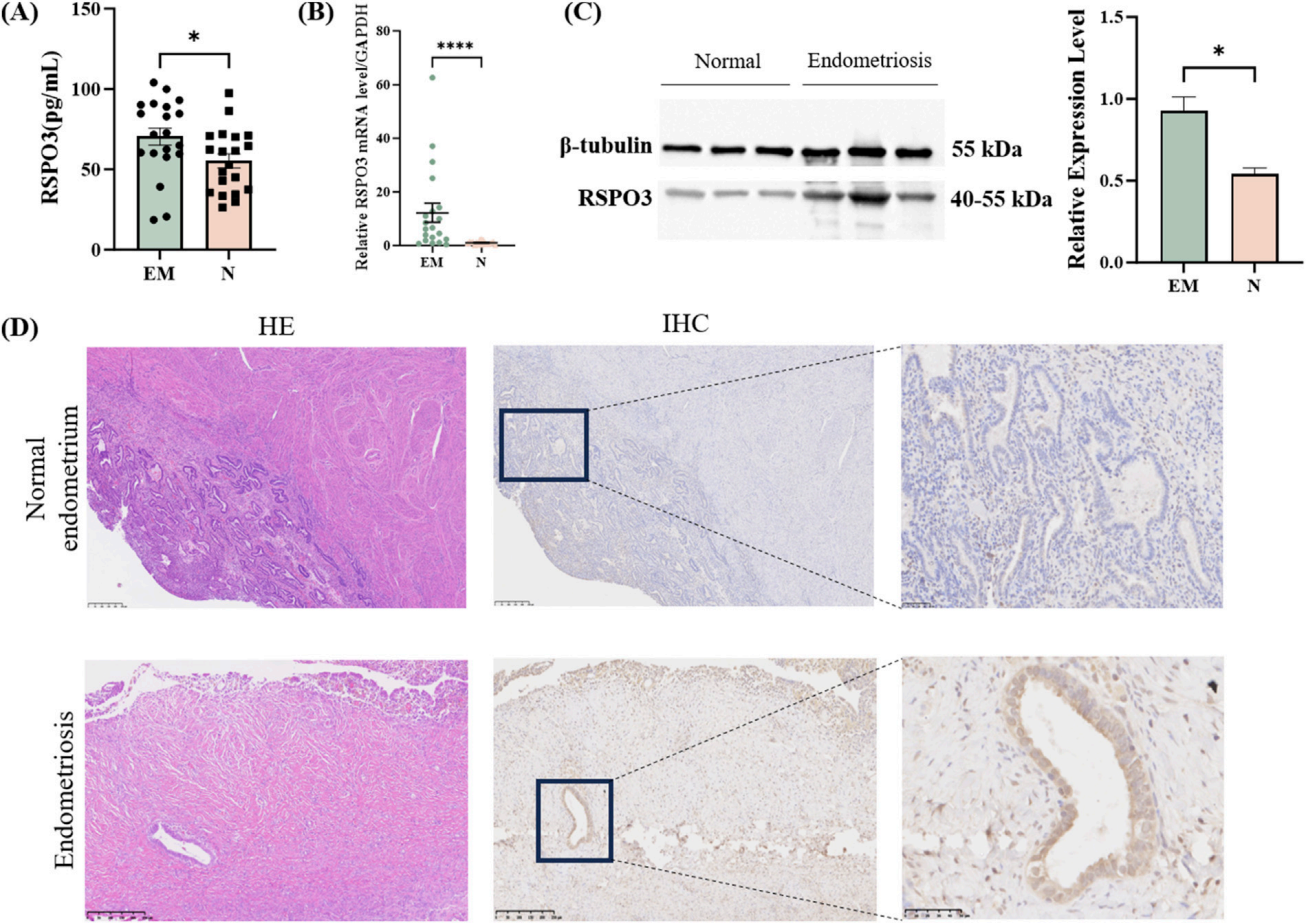
Experimental verification of key proteins in blood and tissues. **(A)** RSPO3 expression levels detected by enzyme-linked immunosorbent assay in EM and normal groups. **(B)** RT-qPCR of RSPO3 in the EM group and normal group. **(C)** RSPO3 protein expression levels. **(D)** HE and IHC staining results of control group and EM group. All the above results are expressed as mean ± SEM. *, *p* < 0.05; ****, *p* < 0.0001. EM: endometriosis; N: normal endometrium.

After confirming the expression of the key plasma protein RSPO3, we first detected the expression of RSPO3 in patient plasma by ELISA. We observed that the expression of the plasma protein RSPO3 was greater in the EM patients than in the controls (Figure 5A). Next, we further examined the mRNA and protein expression of the target genes in the tissues. The RT-qPCR results revealed that the expression of RSPO3 was significantly increased in the tissues of patients (Figure 5B). Furthermore, WB experiments confirmed the above findings (Figure 5C). Full uncropped blot is available at Supplementary Figure S1. These findings suggest that high RSPO3 expression may be a key factor in the development of endometriosis. In order to properly explore the activation of RSPO3 in endometriosis, we further performed HE staining and IHC staining. HE staining can better detect the structure of endometrial glands, which is helpful for the diagnosis of a normal endometrium and endometriosis. After the samples were clearly available, the original paraffin block sections were used for further IHC staining to examine the expression of RSPO3 in the normal endometrium and EM samples (Figure 5D).

## 4 Discussion

In this study, blood metabolites and plasma proteins were used as exposure factors, and genetic variations were used to discover the causal effects of circulating metabolites and proteins on EM to provide preclinical clues for exploring new therapeutic targets for EM. To ensure the reliability of our results, we used two large-scale GWAS datasets to reduce the bias caused by data sources. Sensitivity analysis and colocalization analysis were used to ensure the stability of the results. In our study, no blood metabolites were found to be potentially associated with EM. However, strong evidence indicates that higher genetically predicted circulating RSPO3 levels are associated with an increased risk for EM.

No metabolites survived the stringent Bonferroni correction for multiple testing, indicating limited evidence for robust associations between the metabolome and EM risk. Several factors may contribute to this observation: (1) Limited statistical power: Given the moderate sample size of our metabolome-wide association study (MWAS), the detection of subtle associations—particularly for low-abundance metabolites—may have been underpowered. Larger cohorts are needed to improve statistical precision. (2) Metabolite stability and variability: Metabolite levels can fluctuate due to diurnal rhythms, dietary intake, or pre-analytical processing differences. Such variability may dilute true associations, especially if sample collection protocols were not standardized across cohorts. (3) Medication-related confounding: EM patients frequently use analgesics, hormonal therapies, or other medications that may systematically alter metabolite profiles. Although we adjusted for general medication use, drug-specific effects could mask or distort true metabolic signatures of EM. Future studies employing longitudinal designs, larger sample sizes, and targeted metabolite panels (e.g., focusing on inflammatory or steroid pathways) may help clarify these relationships.

The differential colocalization patterns between RSPO3 and FLT1 provide important insights into their distinct genetic architectures. While both proteins showed significant MR associations, RSPO3 exhibited strong evidence of shared causal variants (PPH4 > 0.9) compared to FLT1. This suggests that the RSPO3-EM association is more likely to be mediated through direct genetic effects at the protein-encoding locus, whereas the FLT1 association may involve more complex regulatory mechanisms. These findings highlight the value of integrating colocalization analysis with MR studies to better characterize protein-disease relationships.

We identified RSPO3 as a novel genetically supported risk factor for endometriosis. Functionally, RSPO3 enhances vascular stability through non-canonical WNT/Ca^2+^/NFAT signaling (Scholz et al., 2016), regulates tissue morphogenesis via syndecan-4-mediated endocytosis (Ohkawara et al., 2011), and activates angiogenesis through Gαi1/3-dependent Akt-mTOR pathways (Xu et al., 2023). These mechanisms align with its established oncogenic roles: In ovarian cancer, RSPO3 promotes tumor angiogenesis and aggressiveness (Gu et al., 2020); in prostate cancer, it drives invasiveness via EMT (Mesci et al., 2019); in bladder cancer, it synergistically activates Wnt/β-catenin and Hedgehog pathways (Chen et al., 2019); and in lung cancer, it confers therapy resistance through inflammasome-mediated pyroptosis (Li et al., 2024). Critically, the convergence of RSPO3’s core functions in vascular remodeling, tissue invasion, and inflammatory microenvironment formation provides mechanistic plausibility for its role in endometriosis pathogenesis—particularly in ectopic lesion survival, neovascularization, and fibrosis progression. Our genetic evidence, coupled with these conserved biological functions across pathological contexts, positions RSPO3 as a compelling therapeutic target worthy of translational investigation in EM. Regarding FLT1, known as vascular endothelial growth factor receptor-1 (VEGFR1), although it passed initial MR analysis and heterogeneity tests, colocalization analysis did not support a stable genetic association. This discrepancy highlights the importance of robust genetic validation in MR studies, even for biologically plausible candidates like FLT1, which is known to regulate angiogenesis.

The mechanism of endometriosis development is controversial. Although the mainstream theory suggests that retrograde menstruation leads to the appearance of endometrial tissue outside the uterus, the factors that induce tissue implantation are key points discussion in the academic community (Olive and Pritts, 2001). Some scholars have proposed that as a benign disease with a malignant growth pattern, the implantation of endometriotic cells is dependent on the formation of new blood vessels (Rocha et al., 2013). In our study, two of the proteins potentially associated with endometriosis were associated with angiogenesis, which may indicate that neovascularization contributes to the biological process of endometriosis. Interfering with local angiogenesis offers an opportunity for the prevention, improvement, or treatment of pelvic endometriosis (Taylor et al., 2002).

The causal role of RSPO3 in endometriosis positions it as a promising therapeutic target. Emerging clinical strategies include **(1)** Monoclonal antibodies (e.g., Rosmantuzumab): that block RSPO3-mediated Wnt activation in angiogenesis (Salik et al., 2020); and **(2)** Small-molecule inhibitors (e.g., Porcupine, PORCN), which are highly specific and essential for catalyzing the palmitoylation of Wnt ligands, thereby facilitating their secretion and biological activity (Yang et al., 2023). Pharmacological inhibition of PORCN is highly selective for Wnt signalling and significantly inhibits the growth of Wnt-dependent tumours, highlighting its promise as a cancer therapy target (Liu et al., 2022). While tissue-specific delivery remains challenging, recent advances in nanoparticle-mediated pelvic targeting offer potential solutions (Li et al., 2023). Repurposing oncology RSPO3 inhibitors thus offers a strategic approach to accelerate endometriosis drug development.

Previous scholars have used MR, colocalization analysis and other methods to find potential correlations between a variety of plasma proteins and EM (Tao et al., 2024; Chen et al., 2025). Owing to the different sources of GWAS data, the requirements of instrumental variable screening and the stringencies of p-value correction, the results are inconsistent. However, it is worth noting that, regardless of the conditions, RSPO3 consistently shows a potential causal relationship with EM. Therefore, the basic experimental verification of RSPO3 expression in EM patients was carried out in this study. To the best of our knowledge, this is the first basic experimental study demonstrating the association between RSPO3 and EM. Blood samples were collected from patients with ovarian endometriosis and normal controls for ELISA analysis. The results indicated that elevated levels of RSPO3 in plasma are indeed associated with the occurrence of EM. We further investigated the differential expression of RSPO3 mRNA in tissues and detected increased expression in ovarian endometriotic cysts. Additionally, the protein expression of RSPO3 was also increased in tissues from EM patients. IHC staining was used to determine the expression of RSPO3 in the tissues, and its expression level was also different from that in normal endometrial glands. These results suggest that RSPO3 may serve as a new target for drug therapy in EM.

However, there are several limitations in this study. Firstly, MR analysis relies on the assumption of genetic instrumental variables. Although we reduced bias through rigorous screening and sensitivity analyses, there may still be unrecognized factors. Secondly, endometriosis includes other types such as deep infiltrating and peritoneal types, in addition to the ovarian type, which were not experimentally studied in this paper. Finally, although experimental verification supports the role of RSPO3 in endometriosis, its specific molecular mechanism still requires further investigation. Future studies will explore the pathological mechanism of RSPO3 in EM and its potential as a therapeutic target through functional experiments and animal models.

## Data Availability

The original contributions presented in the study are included in the article/Supplementary Material, further inquiries can be directed to the corresponding author.
