# Supplementary material for: New therapeutic targets for endometriosis predicted through mendelian randomization analysis and case-control trials
Source: Front Genet. 2025 Aug 15;16:1631446. doi: 10.3389/fgene.2025.1631446 (PMC12394149; doi:10.3389/fgene.2025.1631446)

S1_raw_images. Full uncropped blots. Images of complete membranes, containing prestained protein ladder (both sides), sample lanes (1-6).

Lane labels: 1-3 for the normal endometrium group (NE), 4-6 for the endometriosis group (EM).

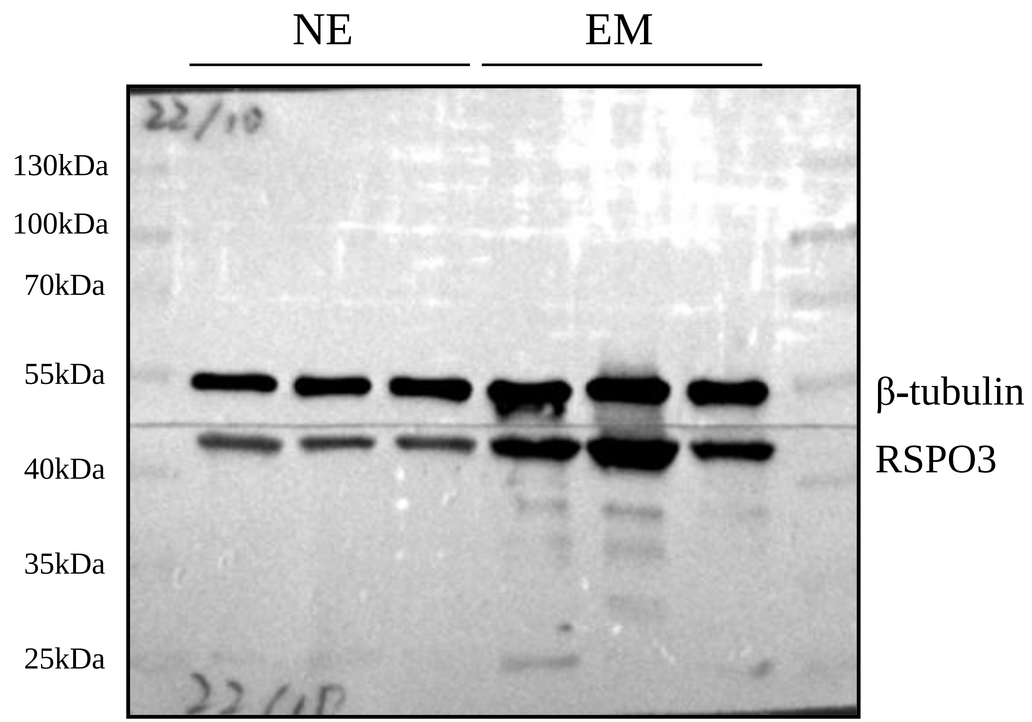

Supplement: Supplementary file 5 [file Image1.pdf]
